# Prevalence of malaria and associated factors among febrile cases attending in Soyama Health Centre, Burji Special Woreda, Southern Ethiopia: a retrospective and an institution-based cross-sectional study

**DOI:** 10.1186/s12936-025-05252-6

**Published:** 2025-02-13

**Authors:** Sintayhu Tsegaye Tseha, Dawit Keshere, Temam Aberar

**Affiliations:** 1 Department of Biology, College of Natural and Computational Sciences, Arba Minch University, Arba Minch, Ethiopia; 2 Soyama Higher Secondary School, Burji Special Woreda, Soyama, Ethiopia

**Keywords:** Burji Special Woreda, Malaria, Prevalence, Associated factors, Trend

## Abstract

**Background:**

Burji special Woreda is one of the malaria endemic areas in Southern Ethiopia. The objective of this study was to determine the prevalence of malaria and associated factors among febrile cases in Burji special Woreda.

**Methods:**

Institutional based cross-sectional study conducted from November 2022 to January 2023. The trend of malaria prevalence was determined based on five years malaria retrospective data (2018–2022). Blood samples were collected from 317 suspected febrile cases to determine the prevalence of malaria in the study area. Thin and thick blood smears were prepared, stained with 10% Giemsa and examined under light microscope. The data on socio-demographic and other determinant factors were collected by interviewers administered pre-tested questionnaire for suspected febrile cases. Bivariate and multivariable logistic regression analysis were done using SPSS software version 20.

**Results:**

Among febrile cases, 22.4% (71/317) were positive for malaria. Being male (P-value = 0.026), living in grass thatched house (P-value = 0.044), availability of mosquito breeding site around residents (p-value = 0.044) and not providing IRS regularly (p-value = 0.008) were significantly associated with *Plasmodium* infection. Based on five years retrospective data (2018–2022), the prevalence of malaria was 27.2% in the study area. The prevalence of malaria showed fluctuating trend between 2018 and 2022 in the study area. Malaria is still prevalent in Burji special Woreda and remains the major public health problems in study area. Interventions against malaria have to be strengthened in order to reduce the burden of malaria in Burji Special Woreda. Furthermore, continuous research on the magnitude of malaria and its associated factors is needed to eliminate the disease from the study area.

## Background

Malaria is the most prevalent mosquito borne diseases throughout tropical and sub-tropical regions of the world with huge medical, economic and social impact [1]. There were an estimated 263 million malaria cases and 597, 000 malaria deaths worldwide in 2023 and Africa accounted for 94% of all malaria cases and 95% of malaria-related deaths. Children under 5 accounted for about 76% of all malaria deaths in the Region [2].

Malaria is a leading public health problem in Ethiopia, where approximately 75% of the land mass of the country has favorable conditions for malaria transmission and 68% population are at risk [3]. In the Southern Nations, Nationalities, and Peoples' Region (SNNPR), malaria occurs in all zones and more than 65% of population being at risk [4]. Burji special Woreda is one of malaria endemic areas in Southern Ethiopia. Based on agro-climatic zones, the Burji Woreda can be divided into three broad climatic zones, namely the highland area of Dega > 2300 m above sea level (m.a.s.l.) which accounts for 21.3% of the total land, the mid-altitude area of Woina Dega and Kolla, between 1500 and 2300 m.a.s.l, which accounts for 42.46% of the total land, and the lowland area of Kolla and Bereha < 1500 m.a.s.l, which accounts for 36.24% of the total land areas of the Woreda. Thus, the aim of the present study was to determine the magnitude of malaria and associated factors among febrile cases in Burji. In addition, the trend of malaria prevalence was assessed based on five years retrospective data obtained from Soyama Health Centre.

## Methods

### Description of the study area

The study was conducted in Burji special Woreda, Southern Ethiopia. The Woreda is bordered with Oromia Region to the East and to the North Amaro Special Woreda and Konso Zone to the South-West. The mean annual rainfall ranges from 801 to 1000 mm while the mean annual temperature from 15.1 to 27.50ºC. The main economic source of the most population of study area is agriculture. According to census, estimated total population of the Woreda was 81,167, of whom 39, 287 are men and 41, 880 women (Ethiopia Statistics Service, 2022). Soyama Health Centre is located in Soyama town, Burji special Woreda at elevation of 1660 m.a.s.l.

### Study design and study period

A cross-sectional study was conducted in Soyama Health Centre to determine the prevalence of malaria and associated factors among febrile cases from November 2022 to January 2023. Five years retrospective data (2018–2022) was also collected from the Soyama Health Center to see the trend of malaria in the study area.

### Study variables

#### Dependent variable

The dependent variable of the study was the test result of malaria parasite in blood.

#### Independent variables

The independent variables of the study are as follows:-

Demographic factors such as age, sex, knowledge about the disease, education level, marital status, occupation, attitude and practice.

Environmental factors include; drainage system, stagnant water, mosquito breeding site, garbage heaps and place of residence.

Household factors include, number of house hold members, and type of house, social economic status, sleeping patterns, and use of insecticide-treated bed nets (ITNs).

### Study population

Febrile patients (age groups ≥ 15) who attend Soyama Health Center during the study period were the study population.

#### Inclusion criteria

The inclusion criteria were being febrile (body temperature of ≥ 38 °C), age ≥ 15 years and being volunteer to give consent to involve in the study.

#### Exclusion criteria

Individuals who were not willing to participate in the study, age below < 15 years and those who were on anti-malarial drug treatment within the last three weeks were excluded. Those who were seriously ill and unable to provide blood samples and Socio-demographic data were also excluded.

### Sample size and sampling procedures

#### Sample size determination

For the cross-sectional study, sample size was calculated using a single population proportion formula at 95% confidence interval, and 5% margin of error (d) = margin of error; (Za/_2_)^2^ standard score (corresponding to 1.96). A previous prevalence of malaria in a study conducted in East Shewa Zone of Oromia Regional State; Ethiopia was 25% [5]. So, the sample size was calculated as follows:-

$$\text{N}= \left(\frac{\text{Za}}{2}\right)2 \times \frac{p\left(1-p\right)}{{d}^{2}}= \left(1.96\right)2\times \frac{0.25\times 0.75}{{0.05}^{2}}=288$$

Finally, by adding 10% non- response rate, the sample size of the study population was found to be 317.

#### Sampling procedures

The blood samples were collected from 317 suspected febrile cases for cross-sectional study. All the febrile cases above ≥ 15 years who come in outpatient department were first assessed by the clinician and those who have the fever were sent to the laboratory for testing using microscopy in Health Centre. Then, questionnaire was administered to whom blood film was drawn during study period and diagnostic results were reported. The recruitment of the study participants continued until the required sample size for each was complete.

#### Data collection, quality control and data analysis

Data were collected through using interviewer administered questionnaire technique. The interviewer administered questionnaire was formulated based on published literatures. Four data collectors, who are health professionals and experienced on malaria services in Soyama Health Centre, collected the data. The principal investigator performed immediate supervision on a daily basis. Each completed questionnaire was checked for completeness. The questionnaires contain five parts. Part A: socio-demographic characters, Part B: knowledge of respondents, Part C: attitude, Part D: environmental factors and Part E: house hold factors. A format which was prepared on a computer spreadsheet (Excel) was used to collect the secondary data (retrospective data, 2018–2022) from Soyama Health Centre record books.

Finger prick blood was collected from malaria suspected adults. A thick and thin blood film was prepared on the same slide [6]. Slides were labelled and air-dried horizontally on a slide tray and thin blood films were fixed with methanol after drying and both thick and thin slides were stained with 10% Giemsa for 10–15 min. The laboratory technician read a total of 100 microscopic areas before declaring a slide negative [7]. Then, parasite positivity was determined from thick smear and species identification was determined from thin smear.

Statistical Package for the Social Sciences (SPSS) version 20 was used for data analysis. Descriptive statistics was done to describe the percentages and frequency distributions of the respondents by socio-demographic variables and others characteristics in the study. Bivariate and Multivariable logistic regression analysis was done to determine the presence and degree of association between dependent and explanatory variables. Odds Ratios (OR) with 95% Confidence Intervals (CI) and p-value < 0.05 was used to determine the presence of statistically significant association between outcome and independent variables. Factors that showed association in bivariate analysis and which has P-value less than 0.25 were included in multivariable logistic regression to control cofounding variables.

### Ethical consideration

The study was conducted after obtaining ethical clearance from the Institutional Review Board of Arba Minch University. Before interview, informed consent was obtained from each study participant.

## Results 

### Socio-demographic characteristics and malaria infection among febrile cases

**Fig. 1:**
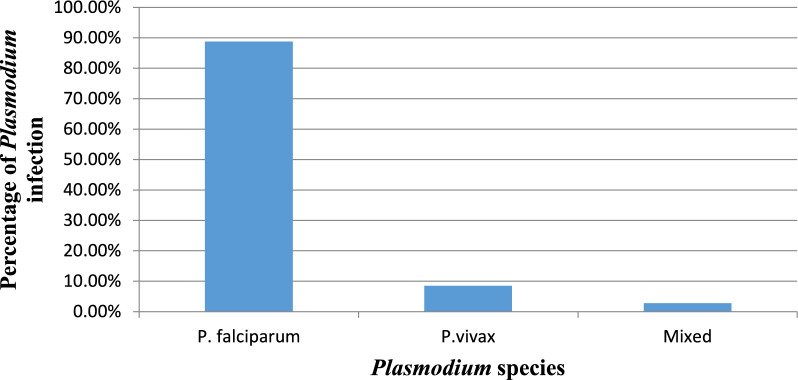
Prevalence of malaria by *Plasmodium* species among febrile cases in Soyama Health Centre, Burji Special Woreda, South Ethiopia Regional State, Ethiopia, 2022/2023

Table 1 and Table 2 show the socio-demographic characteristics and malaria infection among the study participants (Table 1). A total of 317 respondents were included in the study with a response rate of 100%. From the study participants, 71 (22.4%) were positive for malaria, of which 46 (64.8%) were males. In terms of *Plasmodium* species, *Plasmodium falciparum* was the dominant species in the study area (Fig. 1). 262 (82.6%) of respondents were inhabitants of a rural area. Regarding to the respondents’ marital status, 261 (82.3%) were married. In terms of occupation, 165 (52.1%) were farmers and 97 (30.6%) were housewives. The majority of the respondents are illiterates (48.6%). The monthly income of the majority of the respondents’ (89.6%) was less than 1000 Ethiopian Birr.

**Table 1:** Socio demographic variables and malaria infection among febrile cases in Soyama Health Centre, Burji special Woreda, South Ethiopia Regional State,, 2022/2023

Characteristics	N (%)	Positive n (%)	*P. falciparum* n (%)	*P. vivax* n (%)	Mixed n (%)	Negative n (%)
Sex						
Male	151 (47.6)	46 (30.5)	43 (60.6)	3 (4.2)	–	105 (69.5)
Female	166 (52.4)	25 (15)	20 (28.2)	3 (4.2)	2 (2.8)	141 (85)
Age						
15–25	70 (22.1)	12 (17)	12 (16.9)	–	–	58 (83)
25–35	97 (30.6)	21 (21.6)	20 (28.2)	1 (1.4)	–	76 (78.4)
35–45	72 (22.7)	16 (22.2)	13 (18.3)	3 (4.2)	–	56 (77.8)
≥45	78 (24.6)	22 (28.2)	18 (25.4)	2 (2.8)	2 (2.8)	56 (71.8)
Residence						
Rural	262 (82.6)	65 (24.8)	57 (80.3)	6 (8.5)	2 (2.8)	197 (75.2)
Urban	55 (17.4)	6 (10.9)	6 (8.5)	–	–	49 (89.1)
Marital status						
Single	39 (12.3)	9 (23.1)	9 (12.7)	-	-	30 (76.9)
Married	261 (82.3)	58 (22.2)	50 (70.4)	6 (8.5)	2 (2.8)	203 (77.8)
Divorced and widowed	17 (5.4)	4 (23.5)	4 (5.6)	–	–	13 (76.5)
Level of education						
Illiterate	154 (48.6)	31 (20.1)	25 (35.2)	4 (5.6)	2 (2.8)	123 (79.9)
Primary	113 (35.6)	31 (27.4)	29 (40.8)	2 (2.8)	-	82 (72.6)
Secondary	38 (12)	8 (21.1)	8 (11.3)	-	-	30 (78.9)
Tertiary	12 (3.9)	1 (8.3)	1 (1.4)	-	-	11 (91.7)
Monthly income						
< 1000	284 (89.6)	63 (22.2)	56 (78.9)	6 (8.6)	1 (1.4)	221 (77.8)
1000–3000	20 (6.3)	6 (30)	5 (7)	–	1 (1.4)	14 (70)
> 3000	13 (4.1)	2 (15.4)	2 (2.8)	–	–	11 (84.6)
Occupation						
Farmer	165 (52.1)	44 (26.7)	40 (56.3)	4 (5.6)	0	121 (73.3)
House wife	97 (30.6)	18 (18.6)	14 (19.7)	2 (2.8)	2 (2.8)	79 (81.4)
Student	33 (10.4)	4 (12.1)	4 (5.6)	–	–	29 (87.9)
Traders and labour workers	22 (6.9)	5 (22.7)	5 (7.4)	–	–	17 (77.3)

**Table 2:** Respondents knowledge to the cause, transmission and prevention strategies of malaria in Burji special Woreda, South Ethiopia Regional State, 2022/2023

Variable	Total n (%)	Malaria Positive n (%)	Malaria Negative n (%)
Hear about malaria			
Yes	316 (99.7)	71 (22.5)	245 (77.5)
No	1 (0.3)	–	1 (100)
Malaria is transmissible disease			
Yes	293 (92.4)	58 (19.8)	235 (80.2)
No	18 (5.7)	10 (55.6)	8 (44.4)
I do not know	6 (1.9)	3 (50)	3 (50)
Mode of transmission			
Bite of mosquito	251 (79.2)	45 (17.9)	207 (82.8)
Patient contact	2 (0.63)	–	2 (100)
Drinking dirty water	10 (3.2)	3 (30)	7 (70)
Change of weather	1 (0.3)	–	1 (100)
I do not know	29 (9.2)	18 (62)	10 (34.5)
Others	24 (7.6)	5 (20.8)	19 (79.2)
Where mosquito bread			
Stagnant water	292 (92.1)	57 (18.5)	235 (81.2)
Soil	2 (0.6)	–	2 (100)
Running water	2 (0.6)	–	2 (100)
Do not know	16 (5)	13 (81.3)	3 (18.75)
Flower	5 (1.6)	1 (20)	4 (80)
Malaria is curable disease			
Yes	305 (96.2)	67 (22)	238 (78)
No	10 (3.2)	4 (40)	6 (60)
I do not know	2 (0.6)	–	2 (100)
Malaria is preventable			
Yes	302 (95.3)	66 (21.9)	236 (78.2)
No	12 (3.8)	5 (41.7)	7 (58.3)
I do not know	3 (0.9)	–	3 (100)
Prevention and control of malaria			
Sleeping under ITNS			
Yes	283 (89.3)	62 (21.9)	221 (78.1)
No	34 (10.7)	9 (26.5)	25 (73.5)
IRS			
Yes	145 (45.7)	33 (22.8)	112 (77.2)
No	172 (54.3)	38 (22.1)	134 (77.9)
Using medicine			
Yes	249 (78.5)	50 (20.1)	199 (79.9)
No	68 (21.5)	21 (30.9)	47 (69.1)
Environmental management			
Yes	161 (50.8)	40 (24.8)	121 (75.2)
No	156 (49.2)	31 (19.9)	125 (80.1)
Purpose of ITNSs			
Kill mosquitoes	77 (24.3)	8 (10.4)	69 (89.6)
Prevent the entry of mosquito	232 (73.2)	61 (26.3)	171 (73.7)
Kill malaria and repel mosquito	8 (2.4)	2 (25)	6 (75)

### Knowledge of respondents about malaria

As indicated in Table 2, almost all (99.7%) of the respondents heard about malaria. 293 (92.4%) had knowledge about malaria is transmissible disease. 251 (79.2%) respondents had knowledge that malaria is transmitted by bite of mosquito. 89.3% and 78.5% of the respondents had awareness about sleeping under bed net and using medicine were ways of prevention and control of malaria, respectively. However, some participants had misunderstanding related to transmission of malaria, mosquito breeding site, cure of disease, prevention and control and purpose of Insecticide‐treated nets (ITNs).

### Knowledge of respondents about signs and symptoms of malaria

Table 3 shows knowledge of participants about signs and symptoms of malaria in the study area. Fever, headache and chills were the most frequently mentioned signs and symptoms of malaria. Over 90% of the study participants know as fever, headache and chills are signs and symptoms of malaria. More than 50% of the study participants reported that loss of energy, vomiting, sweating, loss of appetite, and joint pain are sign and symptoms of malaria. However, some participants had misunderstanding related to sign and symptom of malaria infection.

**Table 3:** Knowledge of respondents related to signs and symptoms of malaria in Burji special Woreda, South Ethiopia Regional State, Ethiopia, 2022/2023

Characteristics		Frequency (%)	Malaria Positive n (%)	Malaria Negative n (%)
Fever	Yes	294 (92.7)	66 (22.5)	228 (77.5)
	No	23 (7.3)	5 (21.7)	18 (78.3)
Loss of energy	Yes	209 (65.9)	50 (23.9)	159 (76.1)
	No	108 (34.1)	21 (19.4)	87 (80.6)
Headache	Yes	293 (92.6)	70 (23.9)	223 (76.1)
	No	24 (7.4)	1 (4.2)	23 (95.8)
Vomiting	Yes	198 (62.5)	50 (25.3)	148 (74.7)
	No	119 (37.5)	21 (17.6)	98 (82.4)
Sweating	Yes	224 (70.7)	53 (23.7)	171 (76.3)
	No	93 (29.3)	18 (19.4)	75 (80.6)
Chill	Yes	291 (91.8)	70 (24.1)	221 (75.9)
	No	26 (8.2)	1 (3.8)	25 (96.2)
Joint point	Yes	184 (58)	56 (30.4)	128 (69.6)
	No	133 (42)	15 (11.3)	118 (88.7)
Loss of appetite	Yes	217 (68.5)	47 (21.7)	170 (78.3)
	No	100 (31.5)	24 (24)	76 (76)

### Attitudes towards malaria

Table 4 demonstrates the attitudes of the respondents about malaria. Almost all of the respondents (99.4%) agreed on seriousness and threat posed by malaria diseases (Table 4). The majority (94.3%) of respondents thought that best way to prevent malaria is to avoid mosquito bite. 67.5% of respondents believed that adults had greater risk of developing malaria and 92.4% believed that it is dangerous if malaria medicine is not taken completely.

**Table 4:** Attitudes of study participants about malaria, Soyama Health Centre, Burji special Woreda, South Ethiopia Regional State, Ethiopia, 2022/2023

Characteristics	No. (%)	Malaria Positive n (%)	Malaria Negative n (%)
Malaria is serious and threating disease			
Agree	315 (99.4)	71 (22.5)	244 (77.5)
Disagree	2 (0.6)	–	2 (100)
Best way to prevent myself from malaria is to avoid mosquito bite			
Agree	299 (94.3)	64 (21.4)	235 (78.6)
Disagree	18 (5.7)	7 (18.9)	11 (81.1)
Sleeping under a mosquito net during night is one way to prevent may self from getting malaria			
Agree	299 (94.3)	66 (22.1)	233 (77.9)
Disagree	18 (5.7)	5 (27.8)	13 (72.2)
Adults are great risk of getting malaria			
Agree	214 (67.5)	54 (25.2)	160 (74.8)
Disagree	103 (32.5)	17 (16.5)	86 (83.5)
One can recover from malaria without any treatment			
Agree	107 (33.8)	33 (30.8)	74 (69.2)
Disagree	208 (65.6)	38 (18.3)	170 (81.7)
I do not know	2 (0.6)	-	2 (100)
It is dangerous if malaria medicine is not taken completely			
Agree	293 (92.4)	71 (24.2)	222 (75.8)
Disagree	24 (7.6)	–	24 (100)
Go to health centre to blood test as soon as I suspect I have malaria			
Agree	297 (93.7)	70 (23.6)	227 (76.4)
Disagree	20 (6.3)	1 (5)	19 (95)

### Environmental factors associated with *Plasmodium* infection

Table 5 presents environmental factors that are associated *Plasmodium* infection in the study area. 157 (49.5%) of the respondents had mosquito breeding site in their residents. 274 (86.4%) of the study participants mentioned that IRS service was not provided in this village (Table 5).

**Table 5:** Environmental factors with respect to the distribution of malaria parasites among febrile cases in Soyama Health Centre, Burji special Woreda, South Ethiopia Regional State, 2022/2023

Characteristics	Frequency n (%)	Malaria Positive n (%)	Malaria Negative n (%)
Mosquito breeding site in residents			
Yes	157 (49.5)	52 (33.1)	105 (66.9)
No	160 (50.5)	19 (11.9)	141 (88.1)
Good water drainage system			
Yes	91 (28.7)	31 (34.1)	60 (65.9)
No	226 (71.3)	40 (17.7)	186 (82.3)
Clear stagnant water			
Yes	96 (30.3)	26 (27.1)	70 (72.9)
No	221 (69.7)	45 (20.4)	176 (79.6)
Location of residents			
Close to swamp	94 (29.7)	31 (33)	63 (67)
Not close to swamp	223 (70.3)	40 (17.9)	183 (82.1)
Indoor Residual Spraying (IRS) service provided in your village			
Yes	42 (13.2)	18 (42.8)	24 (57.2)
No	274 (86.4)	53 (19.3)	221 (80.7)
I do not know	1 (0.3)	–	1 (100)

Table 6 shows the household factors of respondents that are associated with *Plasmodium* infection. Almost half of the study participants 186 (58.7%) live far from health center, of which 40 (12.6%) positive for malaria. The majority of respondents house were made of mud 165 (52.1%). Higher percentage of malaria positive cases 47 (14.8%) were lived in grass thatched house 117 (36.9%) (Table 6).

**Table 6:** Determinant household factors of the study participants in Soyama Health Centre, Burji special Woreda, South Ethiopia Regional State, 2022/2023

Characteristics	No. (%)	Malaria Positive n (%)	Malaria Negative n (%)
Distance from health facility			
< 1 km	131 (41.3)	31 (23.7)	100 (76.3)
> 1 km	186 (58.7)	40 (21.5)	146 (78.5)
Structure of house			
Grass thatched	117 (36.9)	47 (40.2)	70 (59.8)
Made up of mud	165 (52.1)	18 (10.9)	147 (89.1)
Semi-permanent	35 (11)	6 (17.1)	29 (82.9)
Mosquito nets			
Yes	213 (67.2)	51 (23.9)	162 (76.1)
No	104 (32.8)	20 (19.2)	84 (80.8)
Number of ITNs			
0	108 (34.1)	21 (19.4)	87 (80.6)
1	65 (20.5)	11 (16.9)	54 (83.1)
2	107 (33.8)	27 (25.2)	80 (74.8)
3	37 (11.7)	12 (34.4)	25 (67.7)
House member sleep under mosquito et			
Yes	160 (50.5)	39 (24.4)	121 (75.6)
No	157 (49.5)	32 (20.4)	125 (79.6)
Number of bed suitable for hanging of ITNs			
> 2	77 (24.3)	18 (23.4)	59 (76.6)
2	49 (15.5)	6 (12.2)	43 (87.8)
1	53 (16.7)	10 (20.4)	43 (79.6)
0	138 (43.5)	37 (26.8)	101 (73.2)

### Factors associated with *Plasmodium* infection

Table 7 shows bivariate and multivariable logistic analysis. The variables that have p-value less than 0.25 in bivariate analysis were included in multivariable analysis. The multivariable logistic regression indicated that being male compared to female, grass thatched house compared to semi-permanent, residents live in mosquito breeding site compared to no breeding mosquito site and IRS not provided regularly compared to provide regularly were significantly associated for *Plasmodium* infection. Multivariable logistic regression analysis indicated resident living in a rural area compared to living in an urban area are two times more likely to be infected with *Plasmodium* parasite. Being male (AOR = 2.031, at 95% CI (1.090–3.786) at P-value = 0.026, grass thatched house (AOR = 2.898, at 95% CI, 1.030–8.154) at P-value = 0.044, mosquito breeding site (AOR = 2.035, at 95% CI, (1.019–4.062) at p-value = 0.044 and not providing IRS regularly (AOR = 0.305 at 95% CI, 0.127–0.729) at p-value 0.008 were significantly associated with *Plasmodium* infection (malaria) (Table 7).

**Table 7:** Bivariate and multivariable logistic regression analysis for the factors associated with *Plasmodium* infection in Soyama Health Center, Burji special Woreda, South Ethiopia Regional State, 2022/2023

Explanatory variable	Malaria test		COR (95% CI interval)	AOR (95% CI interval)
	Positive	Negative		
Sex				
Male	46 (30.5)	105 (69.5)	2.471 (1.428–4.277)	2.031 (1.090–3.786)^*^
Female	25 (15.1)	141 (84.9)	1	1
Age of interview				
15–25	12 (17)	58 (83)	1	1
25–35	21 (21.6)	76 (78.4)	1.336 (0.608–2.935)	1.291 (0. 219–3.113)
35–45	16 (22.2)	56 (77.8)	1.381 (0.600–3.179)	0.895 (0.346–2.314)
≥45	22 (28.2)	56 (71.8)	1.899 (0.859–4.198)	1.857 (0.754–4.574)
Residence				
Rural	65 (24.8)	197 (75.2)	2.695 (1.103–6.581)	1.992 (0.728–5.451)
Urban	6 (10.9)	49 (89.1)	1	1
Structure of house				
Grass thatched	47 (40.2)	70 (59.8)	3.245 (1.251–8.421)	2.898 (1.030–8.154)*
Made up of mud	18 (10.9)	147 (89.1)	0.592 (0.216–1.619	0.51 (0.172–1.507)
Semi-permanent	6 (17.1)	29 (82.9)	1	1
Mosquito breeding site				
Yes	52 (33.1)	105 (66.9)	3.675 (2.052–6.584)	2.035 (1.019–4.062)*
No	19 (11.9)	141 (88.1)	1	1
IRS service provided regularly				
Yes	18 (42.9)	24 (57.1)	1	1
No	53 (24)	221 (76)	0.32 (0.162–0.632)	0.305 (0.127–0.729)
I do not know	-	1 (100)	0.000	0.000

**Key**: 1 reference group, *Variables significant at P < 0.05

### Trend of malaria prevalence in Soyama Health Centre

Based on a health center record analysis of malaria over five years, a total of 8064 blood films were diagnosed. Of these, 2196 (27.2%) were positive for malaria. Regarding identified *Plasmodium* species, *P. falciparum* accounted for 1997 (91%) cases, *Plasmodium vivax* accounted for 163 (7.4%) cases, and mixed infection was reported in 36 (1.6%) cases over the past five years (2018–2022). A fluctuating trend of malaria prevalence was noted in the study area. The prevalence of malaria showed slight decrease in two successive years followed by increase in malaria prevalence in 2021, which later decreased in 2022. The prevalence of malaria was 35.8% in 2018, 26.2% in 2019, 26.33% in 2020, 29.4% in 2021, and 20.54% in 2022 (Table 8).

**Table 8:** Trend of malaria prevalence among adults from 2018 and 2022 in Burji special Woreda Soyama Health Centre, South Ethiopia Regional State, 2022/2023

Characteristics	Year					
	2018	2019	2020	2021	2022	Total
Tested for malaria (No)	1334	1644	1591	1723	1772	8064
Malaria Positive No (%)	477 (35.8)	430 (26.2)	419 (26.3)	506 (29.4)	364 (20.5)	2196 (27.2)

## Discussion

This study investigated the prevalence of malaria and associated factors among febrile cases in study area. The findings of this study showed that prevalence of malaria was 22.4% among febrile cases in the study area, which is comparable with recent studies carried out in Mizan Tepi (20.7%) [8] and Enor-Ener Woreda (21.1%) [9]. However, the result of this study is not in agreement with report from Heben Arsi district (6.7%) [10], East Shewa Zone of Oromia Regional State (25.2%) [11] and Northwest, Ethiopia [12].

*Plasmodium falciparum* was dominant species 63 (88.7%) in the study, which is consistent with the findings of Ebabu [9] and Fekadu et al*.* [13]**,** which also reported higher prevalence of *P. falciparum* malaria in Enor-Ener Woreda, Southern Ethiopia and in Dambia district North West Ethiopia, respectively. However, the finding of this study contradicts with studies conducted in other parts of Ethiopia, where *P. vivax* was the dominant *Plasmodium* species [14–16].

Multivariable analysis indicated that males are two times more likely to be infected with malaria parasite as compared with females. This finding is supported by previous studies conducted in other parts of Ethiopia [14, 17, 18]. The higher prevalence of malaria in males might be due to the fact that males have higher outdoor activity and greater occupational risk of being infected with malaria parasites.

The prevalence of malaria among febrile cases living in rural areas was higher than urban residents. The results of this study indicated that residents living in rural areas are two times more likely to be infected with malaria parasite as compared with those living in urban areas. This finding is consistent with report of a study conducted in Dilla town and surrounding rural area, where malaria was prevalent in rural residents [15]. The reason for the difference may be working condition, poor housing condition or weak implementation of malaria prevention activities in rural area.

The prevalence of malaria was higher among illiterates and who had primary education when compared with secondary and tertiary level. The finding of this research is comparable with study conducted in Mizan Aman, Southwest Ethiopia where prevalence of malaria was higher among illiterate than literate [19]. The reason for the difference may be due to differences in the level of understanding of the preventive and control methods of malaria among the study participants. In reference with occupation, the highest prevalence of malaria was observed in farmers and house wives. These findings are in agreement with results of a study conducted in North West Ethiopia, which also reported higher prevalence of malaria among farmers and housewives [14]. The higher prevalence of malaria among individuals with lower income observed in this study is similar to the report of a study that was done in Mizan Aman [19]. This might be due to the fact that individuals with low income do not give attention to prevent transmission of malaria, rather they focus on basic needs.

In the current study, almost all 316 (99.7%) of the respondents had heard about malaria and recognized it as a serious health problem, which is in agreement with previous studies carried out in North West Ethiopia [12]. The majority of the study (79.2%) participants were aware of the fact that the malaria parasites are transmitted by the bite of mosquito, which is consistent with reports from different parts of Ethiopia [12, 20, 21].

The majority of the study participants know that malaria was curable and preventable disease, which is consistent with study carried out by Yhdego et al*.* [20]. Sleeping under mosquito net, using medicine, IRS and environmental management were the main types of malaria preventive measures reported by the study participants. This is in line with previous reports from Chagni health centre, Northwest Ethiopia [12]. With regard to knowledge of malaria intervention measures for prevention and vector control, 283 (89.3%) participants understood ITN as malaria preventive method.

This study showed that 315 (99.4%) respondents know the fact that malaria is dangerous disease and understand the importance of treatment seeking practices. Majority of the respondents reported as they go to health centres when they experience symptoms related with malaria, which is consistent with the finding of Belay et al*.* [12]. In this study, majority of the respondents (94.3%) had a positive attitude towards the benefits of sleeping under ITNs and sleeping under MN is believed to be the best way to prevent malaria infection. This finding is not in agreement with result of a study conducted in Maygaba town, Northwest Ethiopia [20].

In this study, respondents living in Grass thatched houses were found to be 2.9 times more likely to be infected with malaria parasites. The finding is comparable with the result of another study conducted in Ethiopia, which also reported positive relationship between malaria prevalence and living in Grass thatched house [10]. The reason for this relationship might be the fact that grass thatched house has openings that allow mosquitoes to enter.

The results of this study showed that the prevalence of malaria was higher among study participants that were living around mosquito breeding sites. This finding is in agreement with the report of another study that was conducted in Ethiopia [8, 13, 15].

In the study area, houses of the majority of the respondents (86.9%) were not sprayed with IRS, which contradicts with the finding of a study conducted in Northwest Ethiopia [12], where indoor residual spraying (IRS) coverage was 99%. This may explain the reason for the higher prevalence of malaria in Burji Special Woreda as compared with the prevalence of malaria in Northwest Ethiopia.

A fluctuating trend of malaria prevalence was observed in the study area. The prevalence of malaria increased in 2021 in Burji Special Woreda, which decreased in 2022. The finding is comparable with the report of Ebabu who observed fluctuating trend of malaria in Enor Ener Woreda [9]. The increase in malaria prevalence in 2021 Burji special Woreda might be due to weakness in malaria control and prevention interventions following COVID-19 pandemics. The overall percent malaria cases detected in the retrospective malaria study was 27.2% and the most prevalent *Plasmodium* species was *P. falciparum* (91%). The high prevalence of malaria noted in the retrospective study (27.2%) area proved that efforts that have been made to prevent malaria in the Enor-Ener Woreda were not sufficient.

## Limitations of the study

This study is not without limitations. The following limitations were identified: (1) the data was collected only from health facility and this might underestimate the actual burden of malaria in the study area; and (2) the study was conducted during the major malaria transmission season. The findings, therefore, do not include the prevalence of malaria in minor malaria transmission season.

## Conclusion

Based on the results obtained from this study, the following conclusions could be drawn:-

Malaria is one of the major public health problems in the study area with prevalence of 22.4% among febrile cases between November 2022 and January 2023. The retrospective data analysis showed a fluctuating trend of malaria prevalence in the study area. *P. falciparum* is the dominant *Plasmodium* species in the study area. Gender, proximity of residence to mosquito breeding site, structure of the house and irregular use of IRS are the most dominant risk factors for infection with malaria parasite in the study area. Therefore, surveillance of the magnitude of malaria and its risk factors in the study area is needed in order to eliminate the disease from Burji Special Woreda. Furthermore, interventions against malaria have to be strengthened (especially environmental management and regular use of IRS have be strengthened) in order to reduce the burden of malaria in Burji Special Woreda.

## Data Availability

I will provide the research data in request.

## Abbreviations

AOR: Adjusted odd ratio
COR: Crude odd ratio
CI: Confidence interval
ITNs: Insecticide treated nets
IRS: Indoor residual spray
SNNPR: Southern Nations, Nationalities and Peoples’ Region
